# Is CLL curable?

**DOI:** 10.1002/hem3.70308

**Published:** 2026-02-24

**Authors:** Nitin Jain, Alessandra Ferrajoli, Susan O'Brien, William Wierda

**Affiliations:** ^1^ Department of Leukemia The University of Texas MD Anderson Cancer Center Houston Texas USA; ^2^ Division of Hematology/Oncology, Department of Medicine Chao Family Comprehensive Cancer Center, University of California Irvine Medical Center Orange California USA

Historically, the hematologic malignancy, chronic lymphocytic leukemia (CLL), was considered treatable but incurable.^1^ However, this was in the context of treatment with chemoimmunotherapy, and as such, in the era of targeted therapies, we need to reconsider the concept of cure in CLL.^2^

At first, let us review how we might define cure in CLL. There is no established definition of cure in CLL, but extrapolating from other cancers, a reasonable definition would be long‐term remission lasting greater than 10 years in the absence of ongoing treatment. However, an important point to discuss here is what is meant by remission? Does this mean clinical remission, where patients have no symptoms attributable to CLL, blood counts are normal, and there are no palpable lymph nodes versus remission by iwCLL response criteria utilizing bone marrow biopsy and CT imaging studies versus undetectable measurable residual disease (U‐MRD) remission. And if we are talking about long‐term U‐MRD remission, what is the optimal cutoff, traditional 10^−4^ sensitivity versus next‐generation sequencing (NGS) MRD with sensitivity of 10^−6^. As we make the definition of remission stricter, an increasingly lower number of patients will be in the “cure” category 10+ years down the line. U‐MRD remission has emerged as a major determinant of long‐term remission after time‐limited approaches, historically with chemoimmunotherapy and more recently, with venetoclax‐based time‐limited approaches.^3^, ^4^, ^5^ Several studies have shown that achievement of U‐MRD remission (most studies have evaluated U‐MRD4 remission) at the end of treatment leads to improved progression‐free survival (PFS) versus those who remained MRD+, especially for patients with unmutated immunoglobulin heavy chain variable (IGHV) CLL.^3^, ^4^, ^5^

Another important consideration is what is considered a cure in the eyes of the patients. From a patient's perspective, especially for younger patients, “cure” likely means a therapy that is given for a time‐limited duration and leads to lifelong disease control without any chance of disease recurrence. This is a common question in the clinic where patients inquire if a cure is possible in CLL with the available therapeutic options. Older adults (such as patients in their 80s) may have a different expectation of what “cure” means; living their normal life span without CLL‐associated symptoms may be the goal for many such patients.

Historically, two strategies were thought to have the potential for cure of CLL, at least in a subset of patients: (1) FCR (fludarabine, cyclophosphamide, and rituximab) chemoimmunotherapy for young patients with IGHV‐mutated CLL without del(17p)/*TP53* mutation,^6^, ^7^ and (2) allogeneic stem cell transplantation (allo‐SCT).^8^ In a study from our group, among young patients with IGHV‐mutated CLL (*n* = 88), 48.7% were alive and progression‐free after 15 years of follow‐up.^7^ Though only 4 of 45 patients (9%) with IGHV‐mutated CLL progressed beyond 10 years, this, however, indicates that progression can occur after 10+ years even among this favorable genomic subset. In a recent unpublished analysis from our group (manuscript under review, 2025), we evaluated NGS MRD in peripheral blood in patients who had IGHV‐mutated CLL and were in long‐term remission after first‐line FCR. Among 8 patients with available samples (median time from FCR of 12.5 years), only 3/8 were in U‐MRD6 remission (the remaining 5 patients had an NGS MRD detectable CLL clone), despite all 8 patients being without clinical evidence of disease progression. The longitudinal trajectory and clinical significance of these low‐level clones are not clear, but this certainly indicates the presence of CLL in patients assumed to be “cured” of their CLL. Given the risk of therapy‐related myelodysplastic syndromes/acute myeloid leukemia with chemoimmunotherapy and several randomized studies reporting improved PFS/overall survival with targeted therapies compared to that seen with FCR, we do not recommend the FCR regimen for any patient with CLL.^2^, ^9^, ^10^, ^11^ Similarly, given the potential risks involved with allo‐SCT, the safety/efficacy of targeted therapies, the older age group, and comorbidities of patients with CLL, allo‐SCT is used relatively infrequently for these patients.^8^

The field of CLL has moved away from chemoimmunotherapy to targeted therapy in the last decade.^2^ This initially started with the approval of the Bruton tyrosine kinase (BTK) inhibitor ibrutinib for relapsed CLL in February 2014.^12^ Subsequently, several additional BTK inhibitors, namely acalabrutinib, zanubrutinib, and, more recently, pirtobrutinib, were approved for patients with CLL.^13^, ^14^, ^15^ Additionally, the BCL‐2 inhibitor venetoclax is approved for patients with CLL.^16^ CD20 antibody, such as obinutuzumab, is also a well‐established therapeutic modality for patients with CLL and is more effective than rituximab in randomized trials.^5^, ^17^ More recently, a CD19 chimeric antigen receptor (CAR) T cell therapy was approved for relapsed/refractory (R/R) CLL.^18^ These therapies, especially venetoclax‐based combination targeted therapies, are reporting high rates of U‐MRD remissions, including U‐MRD6 remissions by NGS MRD assays.^10^, ^11^, ^19^, ^20^, ^21^, ^22^, ^23^, ^24^, ^25^ It is likely that a subset of patients who achieve early deep MRD responses may remain in ongoing remission long‐term without the need for additional therapy (“cure”). However, we do not yet have 10‐year+ follow‐up for any of the time‐limited venetoclax‐based combination approaches (such as venetoclax + obinutuzumab; venetoclax + BTK inhibitor ± obinutuzumab).

CD19 CAR T cell therapy has revolutionized the therapeutic landscape of CD19^+^ B‐cell malignancies. One of the earlier studies published in 2011 evaluated CD19 CAR T cell therapy in patients with R/R CLL.^26^ A recent update from the same group reported 10+ years remission, including U‐MRD by NGS in two patients with CLL who had received CD19 CAR T in 2010, alluding to the “cure” potential of CAR T cell therapy.^27^ CD19 CAR T, lisocabtagene maraleucel, was approved for patients with R/R CLL in 2024; however, we do not yet have 10+year follow‐up data from this trial.^18^

Though intuitively it makes sense to consider achievement of a deep remission (such as U‐MRD4 or U‐MRD6) essential for “cure,” this concept needs to be reviewed in the context of whether you need to achieve “cure” of CLL to have a normal life expectancy for an individual patient. In a recent pooled analysis of several first‐line Phase 3 trials with ibrutinib, the survival of patients receiving ibrutinib was the same as the age‐matched general population.^28^ We also know that practically no patient will achieve U‐MRD remission with BTK inhibitor therapy alone. This indicates that these patients have a normal survival despite most of them being in partial remission with detectable MRD disease (thereby not meeting the “cure” definition as we discussed above). This can be considered a “functional cure” where patients are living a normal life span with detectable disease.

With the current therapeutic armamentarium of combination targeted therapies, we are achieving high rates of deep‐level remissions for patients with CLL. Whether these therapies will lead to a cure (or functional cure) in a subset of patients is a very likely possibility. Similarly, CAR T cell therapy has the potential for “cure” in a subset of patients. The therapeutic landscape for CLL has improved remarkably in the last decade, and recent data reporting survival of patients treated with single‐agent ibrutinib being similar to the general population is a testimony to the significant advances that have been made in the field of CLL.

## AUTHOR CONTRIBUTIONS

**Nitin Jain**: Conceptualization; writing—original draft; writing—review and editing. **Alessandra Ferrajoli**: Writing—review and editing. **Susan O'Brien**: Writing—review and editing. **William Wierda**: Writing—review and editing.

## CONFLICT OF INTEREST STATEMENT

Nitin Jain has received research funding from Pharmacyclics, AbbVie, Genentech, AstraZeneca, BeOne, Eli Lilly, BMS, ADC Therapeutics, Cellectis, Adaptive Biotechnologies, Precision Biosciences, Fate Therapeutics, Kite/Gilead, Mingsight, Takeda, Newave, Novartis, Carna Biosciences, Sana Biotechnology, Kisoji Biotechnology, Triarm Therapeutics, Bioheng Therapeutics, Ubix Therapeutics, and Ascentage and has participated in advisory boards and received honoraria from Pharmacyclics, Janssen, AbbVie, Genentech, AstraZeneca, BeOne, Eli Lilly, Nurix, BMS, SERB Pharma, Adaptive Biotechnologies, Kite/Gilead, Cellectis, Autolus, Novalgen. Alessandra Ferrajoli ‐ Research support from BeOne, Lilly, Astra‐Zeneca and Janssen. Susan O'Brien ‐ Research funding from Alliance, Caribou, Nurix, and Regeneron, and has served as consultant to Pharmacyclics, Janssen, AbbVie, AstraZeneca, BeOne, Eli Lilly, Nurix, BMS, Autolus, and Epizyme. William Wierda – Research funding from AbbVie, AstraZeneca, Bristol‐Myers Squibb, Genentech, Gilead Sciences Inc, Janssen Biotech Inc, Juno Therapeutics, Eli Lilly, Pharmacyclics, Nurix Therapeutics, BeOne, and is Chair of National Comprehensive Cancer Network CLL guidelines.

## FUNDING

NJ is supported by the Blood Cancer United Career Development Award. This research is supported in part by the MD Anderson Cancer Center NIH/NCI Cancer Center Support Grant P30 CA016672.

## Data Availability

Data sharing is not applicable to this article as no datasets were generated or analyzed during the current study.
